# MiR-144-3p promotes the tumor growth and metastasis of papillary thyroid carcinoma by targeting paired box gene 8

**DOI:** 10.1186/s12935-018-0550-y

**Published:** 2018-04-04

**Authors:** Chang Liu, Chang Su, Yanchun Chen, Guang Li

**Affiliations:** 1 Department of Radiation Oncology, The First Affiliated Hospital of China Medical University, 155 NanJing North Road, Shenyang, 110000 China; 2 Department of Ultrasound Diagnosis, The Liaoning Province People Hospital, Shenyang, China

**Keywords:** Papillary thyroid carcinoma (PTC), Paired box gene 8 (PAX8), miR-144-3p, Cell cycle progression

## Abstract

**Background:**

Paired box gene 8 (PAX8) is expressed in and indispensable to thyroid development. MiR-144-3p is found dys-regulated in cancers, and it can block the expression of target gens. This study sought to understand the effect of MiR-144-3p in papillary thyroid carcinoma (PTC) as well as the associated mechanisms.

**Materials and methods:**

Real-time PCR, immunohistochemical and Western blot assays were performed to examine the expression of target miRNA and/or genes. CCK-8 and flow cytometry analysis was used to respectively test cell growth, cell cycle progression and apoptosis. Luciferase reporter assay was performed to find out whether miR-144-3p could bind to the 3′ untranslated region of PAX8 or not.

**Results:**

We found that PAX8 decreased in PTC, while miR-144-3p increased in PTC. Over-expression of miR-144-3p promoted the cell viability and cell cycle progression. The expressions of cell-cycle-related genes, cyclin D1, cyclin-dependent kinase 2 and CDC25A were modulated by miR-144-3p. Meanwhile, the presence or absence of miR-144-3p both affected epithelial-mesenchymal transition of PTC by regulating the expression of E-cadherin, N-cadherin and vimentin. Moreover, PAX8 may be a potential direct target of miR-144-3p. Mechanically, the activation of extracellular signal–regulated kinases 1/2, Akt and c-Jun N-terminal kinases may be associated with the tumor-promoting effect of miR-144-3p. In addition, the blockage of miR-144-3p forced the anti-tumor effect delivered by X-ray exposure or paclitaxel.

**Conclusion:**

MiR-144-3p promoted the growth of tumor and the metastasis of PTC by targeting PAX 8. The study provided promising prognosis markers and valuable treatment strategy for PTC.

## Background

Papillary thyroid carcinoma (PTC), as a type of well-differentiated malignant tumor, accounts for approximately 80% of all primary thyroid cancers [1], which arises from the thyroid follicular epithelial cells. PTC is frequently found among women and children [2]. In the early stage of PTC, the symptoms of thyroid benign disease are often shown and such a phenomenon could easily lead to misdiagnosis, alternatively the most effective period may be missed. Various genetic and environmental factors contribute to the occurrence of PTC. Though PTC is highly curable due to the application of standard surgical treatment in combination with proper radioiodine ablation therapy, however, the rate of PTC recurrence remains very high and some patients even die from this condition [3]. Furthermore, the possibility of mortality increases in PTC patients if the tumor becomes surgically inoperable and is resistant to radioiodine. Thus, It is critical to develop accurate risk evaluation methods with the aim of preventing the recurrence of thyroid cancer.

The current diagnosis of PTC may be challenged by specimens with low cellularity. The detection sensitivity of PTC may be increased by using immunohistochemical stains. As a member of paired box (PAX) family, paired box gene 8 (PAX8) is a thyroid specific transcription factor. The PAX family are highly conserved in evolution, and it has indispensable functions in the development progression [4]. The critical role of PAX8 in the thyroid development and organogenesis was first reported in 1998 [5]. Various studies have observed that the expression of PAX8 sharply dropped in PTC [6–9], which is considered as a valuable marker for thyroid carcinoma. Based on these investigations, the strategy of affecting the distribution of PAX8 may be of scientific significance.

MicroRNAs (miRNAs), as small non-coding RNAs, have been the focus of researches for decades due to its vital role in gene regulation [10, 11]. The two mechanisms of miRNAs consist of the mRNA degradation and translation inhibition by binding to the 3′untranslational region (3′UTR) of target mRNAs [12]. Different miRNA expression profiles have been found in tumors and normal tissues, and such a phenomenon suggests the key role of miRNA in tumorgenesis [13, 14]. MiRNAs have critical roles in PTC progression [15, 16]. The phenomenon of abnormal miR-144-3p expression has been reported in colorectal cancer (CRC), nonsmall-cell lung cancer (NSCLC), osteosarcoma (OS) and hepatocellular carcinoma HCC [17–20]. However, very little is currently known about and reported on the effect of miR-144-3p. Therefore, it is interesting to investigate the role and relevant mechanisms of miR-144-3p in PTC, and to explore whether PAX8 expression can be regulated by miR-144-3p or not. This study will show a new regulation pathway in PTC development and provide potential prognosis and treatment targets for PTC.

## Materials and methods

### Tissue sample

Thirty paired tumor and adjacent normal tissue samples (at least 5 cm from the tumor loci) were collected from patients who had received surgical resections at The First Affiliated Hospital of China Medical University from December 2014 to January 2016. Informed written consents were obtained from all recruited patients, and the procedures in this study was been approved by the Ethics Committee of Cancer Institute of The First Affiliated Hospital of China Medical University.

### Immunohistochemical (IHC) assay

The samples were treated with paraformaldehyde (4%) and were incubated in 20% sucrose solution at 4 °C overnight. Paraffin embedded tissue Sections (3–4 μm) were used for IHC analysis. After being deparaffinized in xylene and dehydration with graded ethanol, 3.0% hydrogen peroxide in methanol was incubated with the slides at room temperature for 15 min (min) to block endogenous peroxidase. The immunostaining was carried out using primary mouse monoclonal antibodies against PAX8 (1:20, ab53940, abcam, USA).Then the slides were incubated at 4 °C overnight. The primary antibody was first checked by anti-mouse IgG-horseradish peroxidase (HRP)-labeled polymer (ImmunoDetector HRP, CA), and then incubated with 3-3′-diaminobenzidine (DAB) chromogen. The immunostaining score was recorded according to the following criteria [21]: 0—no staining, 1—weak staining, 2—moderate staining, 3—strong staining by two pathologists in a blinded manner.

### Cell culture and treatment

Human PTC cell line (B-CPAP) was purchased from the Chinese Academy of Sciences (Shanghai, China). RPMI 1640 medium containing 10% newborn bovine serum (FBS) were used for the culture of B-CPAP cells. The culture condition was set at 37 °C with 5% CO_2_. The cell grouping was as follows: Control, untreated PTC cells; mimics, PTC cells transfected with miR-144-3p mimics; inhibitor: PTC cells transfected with miR-144-3p inhibitors; NC, PTC cells transfected with miRNA negative control. For X-ray exposure, according to a previous study [22] irradiation dose was 3 Gy at 1.37 Gy/min at room temperature on an industrial portable at 150 kV X-ray unit (Philips Medical Systems). For paclitaxel treatment, cells were treated with 40 nM paclitaxel for 24 h [23].

### Cell transfection

MiRNA scramble control vector (CmiR0001-MR04), has-miR-144 mimics vector (HmiR0275) and has-miR-144-3p inhibitor vector (HmiR-AN0189) were purchased from Guangzhou FulenGen Co., Ltd. Cell transfection was carried out using Lipofectamine 3000 (L3000008, Invitrogen) according to the manufacturer’s instruction.

### CCK-8 method

The proliferation of PTC cells was determined by CCK-8 method according to the manufacturer’s instructions. At a density of 1 × 10^3^ cells per well, cells were seeded in 96-well plates and transfected. After being transfected for 48 h, cells were then mixed with 10 μl CCK-8 solution (C0038, Beyotime, China) and put into incubation for 4 h at 37 °C. Finally, the optical density at a wavelength of 450 nm was recorded by a microplate reader (Biorad, USA).

### Flow cytometric assay

Cell cycle distribution was determined using a propidium iodide (PI) staining. The cells were starved overnight in order to acquire the synchronizated cells, which were seeded into a 6-well plate at a density of 2 × 10^6^ cells per well. After listed treatments, the cells were collected and fixed with 75% ethanol at 4 °C overnight. The next day, the cells were stained with PI (Sigma-Aldrich) and RNase A (Sigma-Aldrich). Fluorescein isothiocyanate (FITC)-annexin V/PI staining kit was applied (V13241, Invitrogen, USA). The cells were harvested and stained with Annexin V-FITC for 15 min and with PI for 5 min in the dark. The fluorescence signals were collected by FACSCanto (BD Bioscience, San Jose, CA) and then analyzed by FlowJo 8.7.1 software (Ashland, OR).

### Luciferase reporter assay

At a density of 2 × 10^4^ cells per well, PTC cells were cultured in a 96-well plate. 3′UTR-PAX8 firefly luciferase reporter (HmiT018794-MT06) was co-transfected with miR-144-3p mimics or inhibitors by using Lipofectamine 3000. The pRL-TK (Promega, Madison, WI) was seen as normalized control. The mutated (mut)-3′UTR-PAX8 firefly luciferase reporter was generated using a site-directed mutagenesis kit (KM131204, Tiangen, China) according to the manufacturer’s instructions. This mutated vector was treated as negative control. After 24 h, luciferase substrates for firefly and renilla were added into the cell lysate in each group. The Dual-Glo Luciferase Assay System (E2920, Promega) was adopted for measuring luciferase activity on a Turner BioSystems 20/20n luminometer.

### Real-time PCR

Total RNA was isolated using Trizol regent (15596018, Invitrogen, USA) following the protocols. The M-MLV reverse transcriptase (promega, USA) and oligodT (or miRNA first-strand cDNA synthesis kit (Invitrogen)) were employed to synthesis cDNAf from mRNA or miRNA. The quantification of cDNA was performed on 7500-fast RT-PCR System (Applied Biosystems, USA) using SYBR Mixture (CWBio, China). Relative expression levels of U6, β-actin or GAPDH endogenous control were calculated by 2^ − ΔΔCT^ method [24]. The primers used were as follows:

CDK2 forward 5′-CCTGGATGAAGATGGACGGA-3′,

CDK2 reverse 5′-GGAGAGGGTGAGATTAGGGC-3′;

CDC25A forward 5′-ACAACCGATGCAAGCTGTTT-3′,

CDC25A reverse 5′-CTCATGGGCCTTCTCTGGAT-3′;

Cyclin D1 forward 5′-CCCTCGGTGTCCTACTTCAA-3′,

Cyclin D1 reverse 5′-CTTAGAGGCCACGAACATGC-3′;

E-cadherin forward 5′-TCACATCCTACACTGCCCAG-3′;

E-cadherin reverse 5′-AGTGTCCCTGTTCCAGTAGC-3′;

N-cadherin forward 5′-ATATTTCCATCCTGCGCGTG-3′;

N-cadherin reverse 5′-GTTTGGCCTGGCGTTCTTTA-3′;

Vimentin forward 5′-AATAAGATCCTGCTGGCCGA-3′;

Vimentin reverse 5′-GGTGTTTTCGGCTTCCTCTC-3′;

PAX8 forward 5′-GCCTTCTCCCTCTGCCTTTA-3′;

PAX8 reverse 5′-TTATGCAGGCTCCAGTCACA-3′;

Bax forward 5′-GTGCCGGAACTGATCAGAAC-3′;

Bax reverse 5′- CCAAAGTAGGAGAGGAGGCC-3′;

Bcl-2 forward 5′-GCCTTCTTTGAGTTCGGTGG-3′;

Bcl-2 reverse 5′-GAAATCAAACAGAGGCCGCA-3′;

β-actin forward: 5′-CTCCATCCTGGCCTCGCTGT-3′;

β-actin reverse: 5′-GCTGTCACCTTCACCGTTCC-3′;

U6 Forward 5′-CTCGCTTCGGCAGCACA-3′;

U6 Reverse 5′-AACGCTTCACGAATTTGCGT-3′;

GAPDH forward: 5′-CACAGTCCATGCCATCACTG-3′;

GAPDH reverse: 5′-ATCTCGCTCCTGGAAGATGG-3′;

### Western blot

Protein extraction kit (Solarbio, China) was used to isolate proteins, and then the concentration of the proteins was detected by BCA Protein Assay kit (Cwbio, China). The cell lysate was loaded on 10% SDS-PAGE after being boiled for 5 min to denaturation and then transferred onto a PVDF membrane (Millipore, USA). The non-specific antigens were blocked using 5% skimmed milk. Primary antibodies were incubated at 4 °C overnight. After removing the appropriate horseradish peroxidase-conjugated secondary antibody (ab97051, 1:3000, Abcam, USA), the blots were detected using Enhanced Chemiluminescence Detection kit (GE Healthcare Biosciences). The primary antibodies used in the experiment were as follows: N-cadherin (ab18203, 1:1000), Vimentin (ab137321, 1:1500), anti-PAX8 (ab53940,1:200), anti-p-Erk1/2 (ab201015, 1:1000), anti-Bcl-2 (ab593480, 1:700), anti-Bax (ab53154, 1:1000), anti-Bak (ab32371, 1:10,000) purchased from Abcam. E-cadherin (3195, 1:1000), anti-p-Akt (Thr308) (13,038, 1:1000), p-JNK1/2(4668, 1:2000), anti-GAPDH (5174, 1:1000), anti-β-actin (4970, 1:1000) purchased from Cell Signaling Technology.

### Statistics

Student’s t-tests or one-way analysis of variance (ANOVA) was performed to analyze the differences among groups. P < 0.05 was considered statistically significant. The statistical analysis were conducted using GraphPad Prism 5.0 software (GraphPad Software, Inc., La Jolla, CA, USA).

## Results

### The expressions of miR-144-3p and PAX8 in patients diagnosed with PTC

**Fig. 1:**
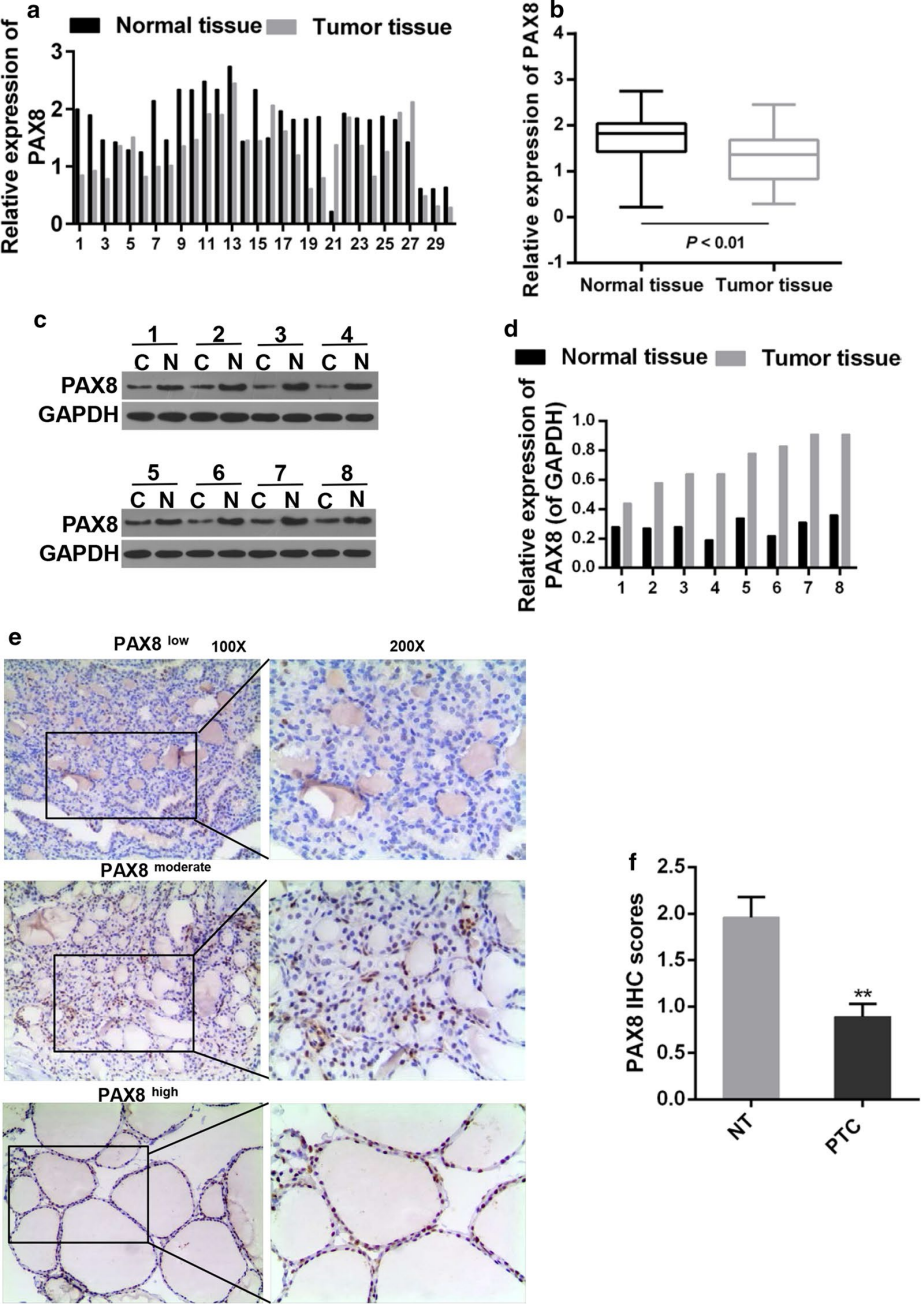
**a** Quantitative analysis was applied for expression level of PAX8 in cancer and adjacent normal tissues. **b** Paired T test was adopted for PAX8 expression between cancer and normal tissues. ***P < 0.001. **c**, **d** Western blot was used for PAX8 expression in 8 randomly selected patients out of the 30 matched initial samples. C, cancer tissues; N, normal tissues (**e**, **f**) IHC analysis for the distribution of PAX8. **P < 0.01 vs. NT. *NT* normal tissue, *PTC* cancer tissue

**Fig. 2:**
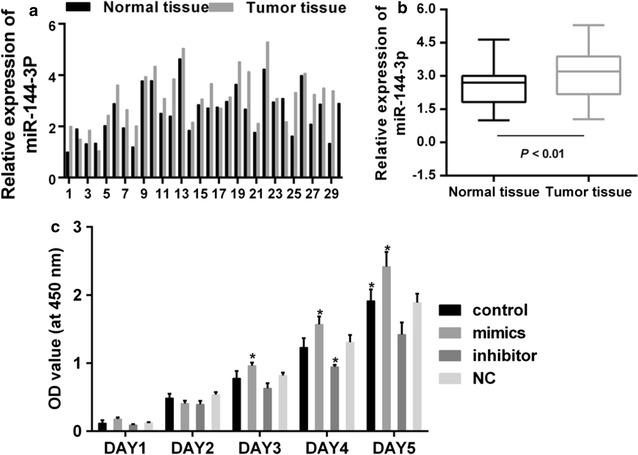
**a** Quantitative analysis was performed for expression level of miR-144-3p in cancer and adjacent normal tissues. **b** Paired T test was carried out for miR-144-3p expression between cancer and normal tissues. ***P < 0.001. **c** Cell viability of PTC cells. *P < 0.05 vs. control

The expressions of miR-144-3p and PAX8 in tumor and the corresponding normal tissues were detected first. The RT-PCR results showed that compared to those in normal tissues, in tumor tissues the expression of miR-144-3p was increased but PAX8 expression was dropped (P < 0.001, Fig. 1a–d). The protein levels of PAX8 in 8 randomly selected PTC patients were examined by Western blot. The results showed that the expression of PAX8 was blocked at the translational level (P < 0.001, Fig. 1e–f), and that the distribution of PAX8 reduced in PTC tissues compared to that of the normal tissues (P < 0.05, Fig. 2a, b).

### MiR-144-3p inhibitors reduced proliferation and induced cell cycle arrest of PTC cells

**Fig. 3:**
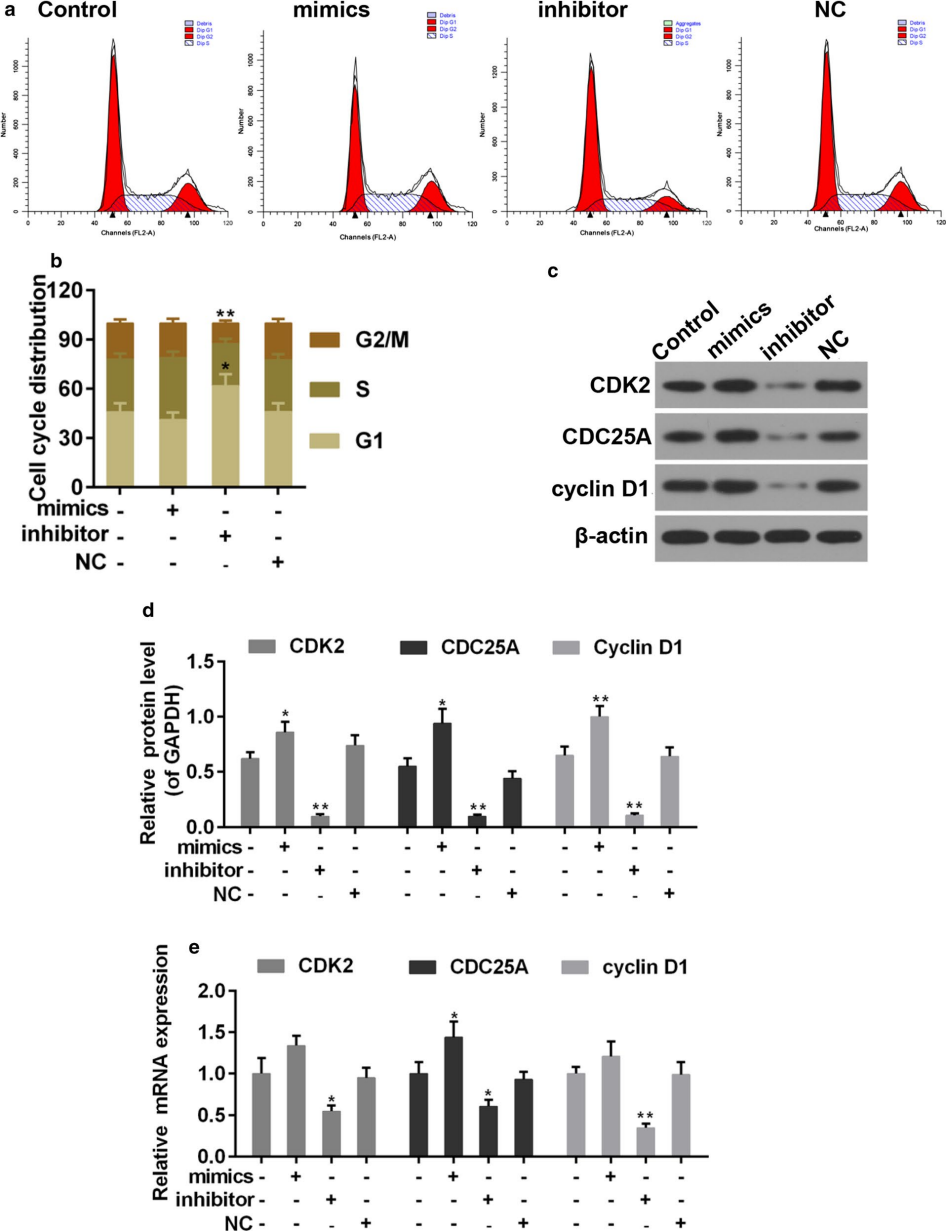
**a**, **b** Flow cytometry analysis was applied for cell cycle distribution. **c**, **d** Western blot was used for expression of CDK2, CDC25A and cyclin D1. **e** Quantitative analysis was used for expression of CDK2, CDC25A and cyclin D1. *P < 0.05 and **P < 0.01 vs. control

Un-controlled growth is a typical character of tumor cells. The cells were treated and grouped as follows: Control, untreated PTC cells; mimics, PTC cells transfected with miR-144-3p mimics; inhibitor: PTC cells transfected with miR-144-3p inhibitors; NC, PTC cells transfected with miRNA negative control. The CCK-8 assay result indicated that the increased expression of miR-144-3p augmented the cell viability of PTC cells, while its suppressed expression reduced the cell viability (Fig. 2c). Researchers pointed out that the cell proliferation was largely dependent on the normal progression of cell cycle [25], therefore, the cell cycle progression was tested (Fig. 3a, b). The results from our tested showed that in miR-144-3p inhibitor group the cell populations at G1 phase increased significantly but decreased largely at G2/M, if being compared to control group. The proportion of cell in S phase was less in miR-144-3p inhibitor group than that those in control group. However, the effect of miR-144-3p mimics was the opposite, although no significant differences was observed. The expression of cell-cycle-related proteins, including CDK2, CDC25A and cycline D1, were also determined. Noticeably, the mRNA and protein expression of these factors was increased by miR-144-3p mimics but decreased by inhibitor (Fig. 3c, d).

### MiR-144-3p modulated the expression of metastasis associated proteins in PTC cells

**Fig. 4:**
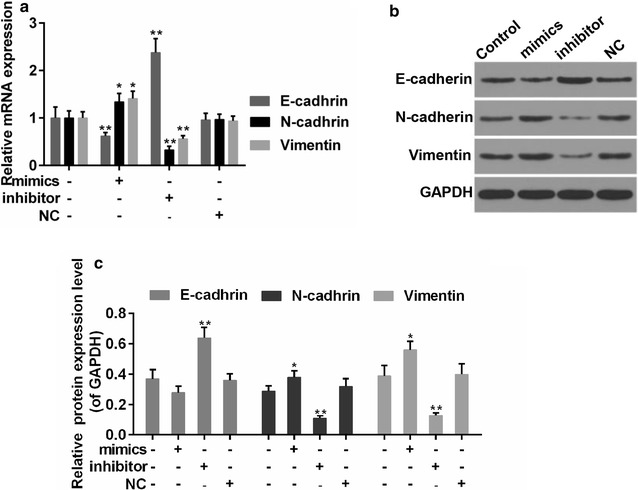
**a** Quantitative analysis was performed for expression of E-cadherin, N-cadherin, and vimentin. **b**, **c** Western blot analysis was carried out for E-cadherin, N-cadherin, and vimentin. *P < 0.05 and **P < 0.01 vs. control

Another feature of cancer cells is the ability of metastasis. Epithelial-mesenchymal transition (EMT) is a primary mechanism responsible for metastasis, which is accompanied with the acquirement of mesenchymal phenotypes and the loss of cell polarity. E-cadherin, N-cadherin and vimentin are the proteins participate in EMT [26]. Thus, we applied RT-PCR and Western blot assays to analyse the expressions of EMT-associated proteins (Fig. 4a–c). Data showed that the expression of E-cadherin was reduced by miR-144-3p, while the expressions of N-cadherin and vimentin were increased by miR-144-3p. By contrast, the expressions of E-cadherin, N-cadherin and vimentin in miR-144-3p inhibitor group were reversed.

### PAX8 was a potential direct target of miR-144-3p

**Fig. 5:**
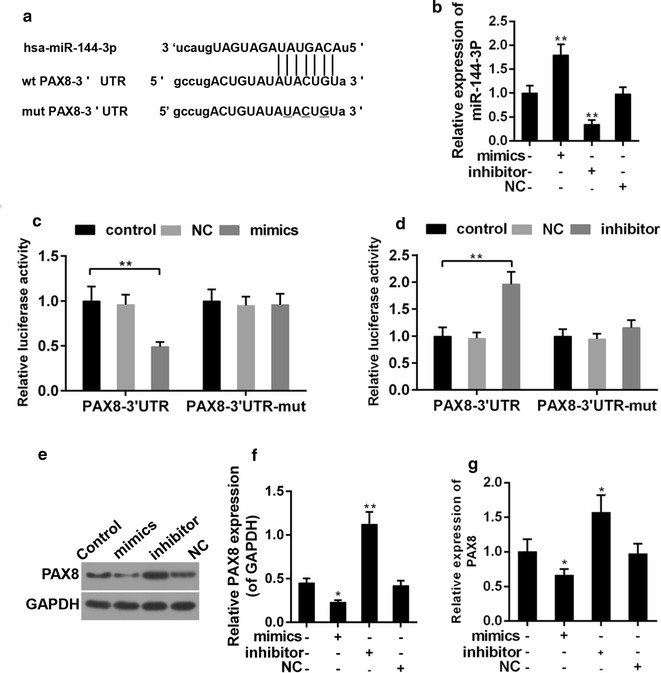
**a** The prediction binding sequence of miR-144-3p in PAX8 mRNA 3′-UTR. wt: wild-type; mut: mutated. **b** Transfection efficiency of miR-144-3p mimics and inhiitors. **c**, **d** Relative luciferase activity of TLR4 mRNA 3′-UTR after transfection with miR-144-3p mimics or inhiitors. **P < 0.01. **e**, **f** The effect of miR-144-3p on the protein expression of PAX8. **P < 0.01 vs. control. **g** The effect of miR-144-3p on the mRNA expression of PAX8. **P < 0.01 vs. control

To understand the mechanisms that underlie the effect of miR-144-3p on PTC cells, the available database TargetScan was adopted to help predict the potential target of miR-144-3p. The data suggested that there were possible binding sites of miR-144-3p in the 3′UTR region of PAX8 (Fig. 5a). Thus, luciferase reporter assay was performed to confirm such a prediction. The transfection of miR-144-3p mimic and its inhibitors was effective in PTC cells (Fig. 5b). Moreover, the luciferase activity of PAX8-3′UTR was markedly inhibited by over-expression of miR-144-3p. Nevertheless, the luciferase activity of PAX8-3′UTR-mut was not affected (Fig. 5c). Furthermore, while miR-144-3p inhibitors enhanced the luciferase activity of PAX8-3′UTR, it produced no effect on that of PAX8-3′UTR-mut (Fig. 5d). To further confirm the effect of miR-144-3p on the translation of PAX8, the expression of PAX8 was determined in the presence of miR-144-3p or the miR-144-3p inhibitors. We observed that the mRNA and protein levels of PAX8 were decreased and forced by miR-144-3p mimics and inhibitors, respectively (Fig. 5e–g).

### MiR-144-3p suppressed the activity of ERK1/2, Akt and JNK signals

**Fig. 6:**
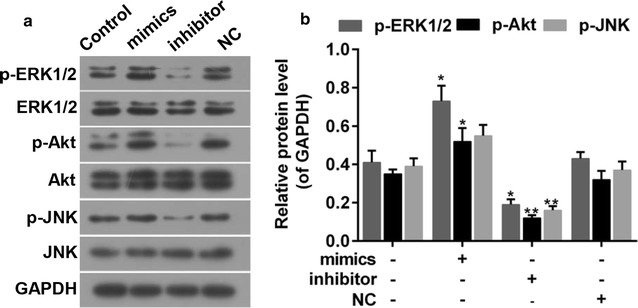
**a**, **b** The effect of miR-144-3p on the activations of ERK1/2, Akt and JNK signals. *P < 0.05 and **P < 0.01 vs. control

The activities of ERK1/2, Akt and JNK were studied in order to further investigate the underlying molecular mechanism. The results showed that the phosphorylation of these signal proteins was enhanced in the presence of miR-144-3p but otherwise declined in the absence of miR-144-3p (Fig. 6a–c).

### Down-regulation of miR-144-3p enhanced the effect of X-ray exposure and paclitaxel in PTC cells

**Fig. 7:**
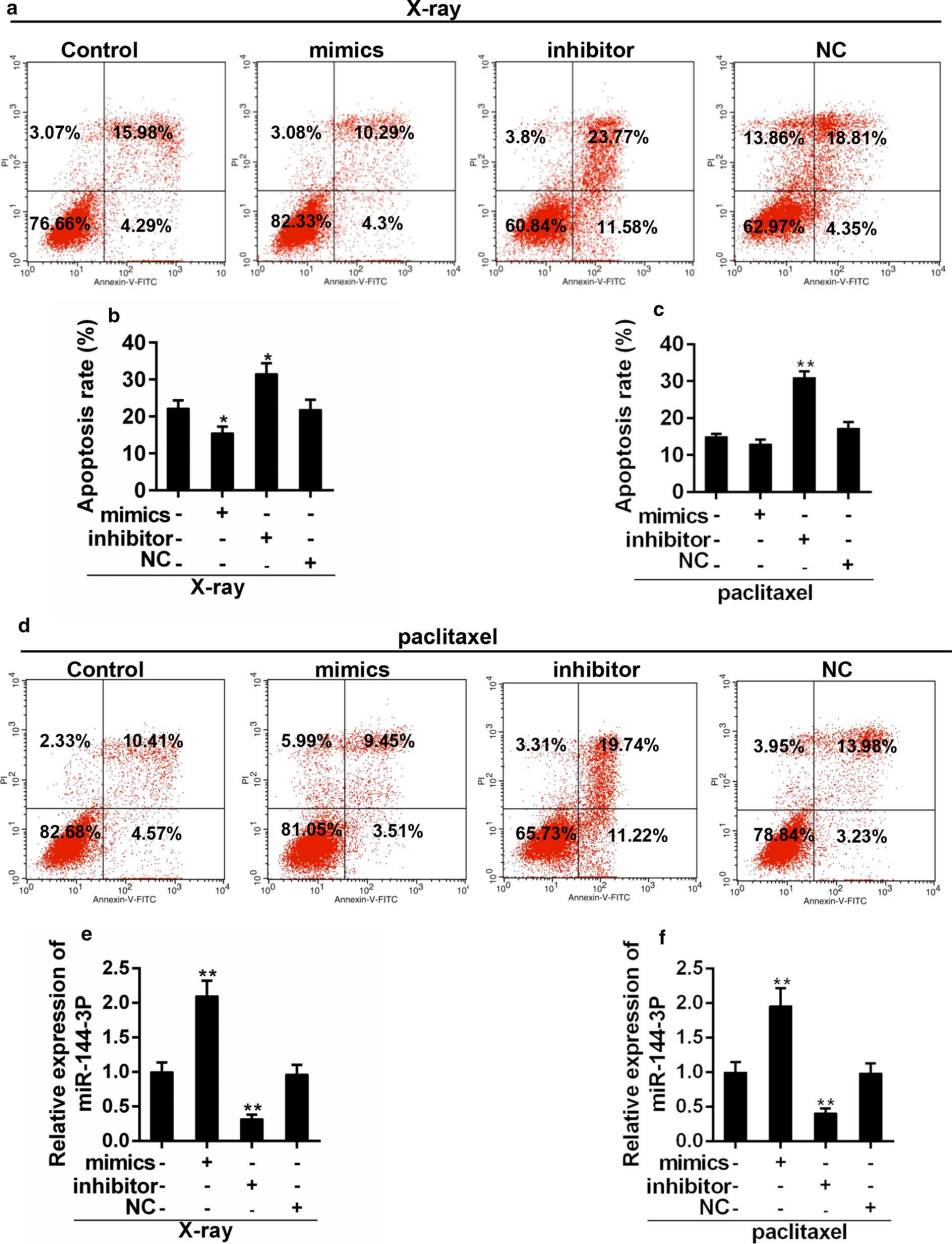
**a**, **b** Flow cytometry analysis for apoptosis of PTC cells treated with X-ray and miR-144-3p. *P < 0.05 vs. control. **c**, **d** Flow cytometry analysis was carried out for apoptosis of PTC cells that were treated with paclitaxel and miR-144-3p. **P < 0.01 vs. control. **e**, **f** Transfection efficiency of miR-144-3p mimics and inhiitors in PTC cells that were treated with X-ray (**e**) or paclitaxel (**f**). **P < 0.01 vs. control

**Fig. 8:**
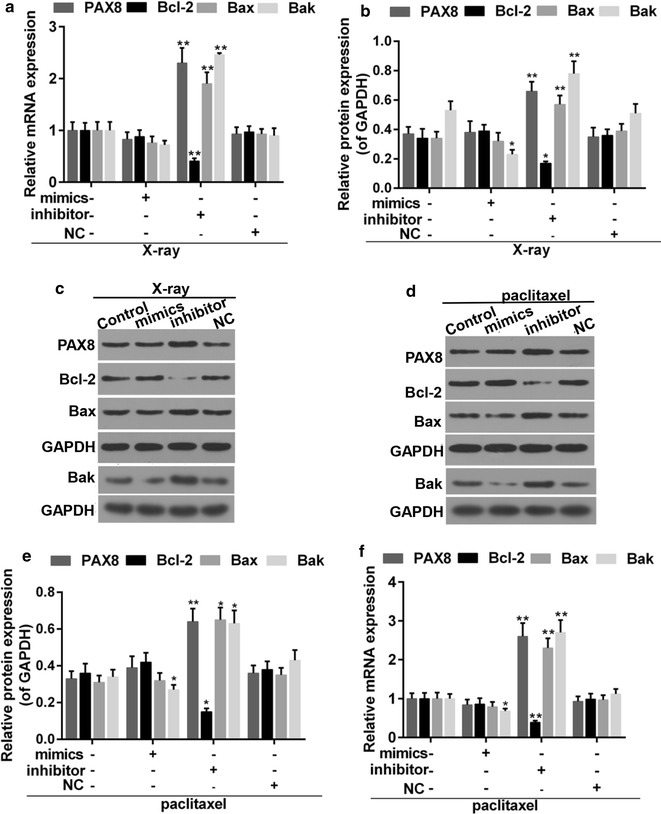
**a** Quantitative analysis was carried out for expressions of PAX8, Bax, Bcl-2 and Bak in PTC cells that were treated with X-ray and miR-144-3p. **b**, **c** Western blot was performed for expression of PAX8, Bax Bcl-2 and Bak. **d**, **e** Western blot was used for expressions of PAX8, Bax Bcl-2 and Bak in PTC cells that were treated with paclitaxel and miR-144-3p. **f** Quantitative analysis was performed for expression of PAX8, Bax Bcl-2 and Bak. *P < 0.05, **P < 0.01 vs. control

X-ray irradiation and paclitaxel are common options for the treatment of cancer. To further confirm the effect of miR-144-3p on PTC, the apoptosis rate of PTC cells, which were treated with appropriate X-ray or paclitaxel, was examined in the presence and absence of miR-144-3p. Our experiment observed that in both the X-ray exposure and paclitaxel treatment group (Fig. 7a–d), apoptosis was depressed by miR-144-3p but induced by its inhibitors. The transfection efficiency of miRNA was demonstrated (Fig. 7e–f). We also found that the expression of PAX8 was inhibited after the transfection of miR-144-3p, and that the expressions of pro-apoptotic proteins, Bax and BAK, were increased by miR-144-3p inhibitors. However, the anti-apoptotic protein, Bcl-2 was depressed in miR-144-3p inhibitor group (Fig. 8a–f). Thus, the down-regulation of miR-144-3p synergistically enhanced the anti-tumor effect of X-ray exposure and paclitaxel.

## Discussion

Accurate stratification and prognostic evaluation play critical roles in terms of reducing the occurrence of PTC. The use of immunohistochemical stains may enhance the diagnostic sensitivity of PTC in cells from thyroid fine-needle aspiration [27]. PAX8 is important and indispensable in the thyroid development. Increased expression of PAX8 was observed in ovarian cancer, bladder, prostate and endometrial carcinomas [28], however, its reduced expression was found in thyroid organogenesis [29]. The study proved the decreased expression of PAX8 both in the transcriptional and translational levels in patients diagnosed with PTC. As a transcriptional factor, the expression of PAX8 target genes in PTC have been studied [30]. Although this study enriches the understanding of the mechanisms that take part in thyroid tumorigenesis, to investigate the upstream regulator of PAX8 may be more urgent and effective in combating t the reoccurrence of PTC.

MiRNAs have been observed participating in the progressive event that associated with complex gene expression and regulation [31]. More and more researchers are interested in studying the role of miR-144-3p in cancer development and treatment. It has reported that the expression of miR-144-3p was depressed in several cancers, such as CRC, OS and NSCLC [17–20]. However, the effect of miR-144-3p on PTC varies from cancers to cancers. In this study, the expression of miR-144-3p showed an increased trend in PTC patients. Such a phenomenon was in line with the up-regulation of miR-144-3p in Clear cell renal cell carcinoma [32]. It was indicated miR-144-3p may have positive role in the PTC carcinogenesis. Results from CCK-8 assay were consistent with this speculation. Unlimited proliferation is a hallmark of cancer, which is caused by the aberrant cell cycle progression [25]. G1/S transition is regarded as a key step during cell cycle, and it promotes cell proliferation [33]. Our results showed that miR-144-3p promoted G1/S transition, however, miR-144-3p inhibitor induced G1/S transition arrest. Cyclins and cyclin-dependent kinases (CDKs) are the important regulatory proteins during cell cycle progression, in which progress CDK2 and cyclin D1 are involved. CDC25A is also able to control G1/S and G2/M transition [34]. Thus, we went further to determine the expression of these transition regulators in order to investigate the cell cycle promotion effect of miR-144-3p. The results revealed that the expression of cyclin D1, CDK2 and CDC25A was forced by miR-144-3p mimics but alleviated by inhibitors. Researches reported that EMT was related to tumor metastasis [35, 36]. The over-expression of miR-144-3p facilitated EMT, which is a consequence of the up-regulation of mesenchymal markers (N-cadherin, and vimentin) and down-regulation of epithelial marker (E-cadherin). Thus, we concluded that miR-144-3p was positive to promote the tumorgenesis of PTC. However, studies have indicated the anti-tumor function of miR-144-3p [37–39]. The distinct effect of miR-144-3p may vary dependent on cell context and microenvironments around.

Luciferase reporter assay was carried out To explore the mechanisms that underlie the role of miR-144-3p in PTC. The results suggested that miR-144-3p repressed the activity of 3′UTR-PAX8, which was enhanced by miR-144-3p inhibitors. In addition, the expression of PAX8 was repressed by miR-144-3p. The results demonstrated that PAX8 expression may be directly regulated by miR-144-3p. Accordingly, miR-144-3p may be a candidate to prevent PTC. To a large extent, biological events are the consequences of signal transduction. ERK1/2, Akt and JNK have been reported by previous studies due to their important roles in living cells, including in cancer cells [40, 41]. Our findings showed that to suppress these signals was at least partly related to the mechanism via which miR-144-3p forced the progression of PTC. The activation of Erk1/2, JNK has been reported in prostate cancer and colon cancer [42, 43], and the activation of ERK1/2 and Akt and inactivation of JNK were found in lung cancer [44]. These findings implied the complexity of the signal cascades in carcinogenesis.

To further test the effect of miR-144-3p, the treatment model was set up in vitro. The cluster formations of Bax and Bak could be induced upon apoptosis stimuli, which exerts pro-apoptotic effect [45]. Bcl-2 is considered as an anti-apoptotic factor [46]. We found that miR-144-3p inhibitor increased the expression of Bax/Bak but decreased the expression of Bcl-2. Such an observation suggested that the the treatment in combination with miR-144-3p inhibitor promoted the sensitivity to apoptosis of PTC,if being compared to treatment with the use of X-ray or paclitaxel only. These results strongly indicated the tumor-promoting function of mir-144-3p and its potential clinical significance in the treatment of PTC. Thus, to continue the studies in vivo should be encouraged.

The sample size of the PTC patients was another limitation of this study. Investigation with larger-scale samples would be more convincing and closer to the reality. In addition, exploring, ipso facto, the regulation mechanisms between miR-144-3p and the signal cascades (ERK1/2, Akt and JNK) was beneficial in understanding the accuracy molecular events that happened during the progression of PTC.

## Conclusion

The current study unveiled the tumor-promoting effect of miR-144-3p in PTC mainly through promoting cell cycle progression and EMT. Our findings also suggested that PAX8 may be a direct target of miR-144-3p, and that the effect of miR-144-3p may be partially related to the activations of ERK1/2, Akt and JNK signals. In addition, the blockage of miR-144-3p could synergistically force the effect of X-ray exposure and paclitaxel. To conclude, this study provided promising prognosis markers and valuable treatment strategy for PTC.
